# Polymorphism of the IL-18 and CD40 genes and Liver Transplant Outcome in Iranian Patients

**Published:** 2014-11-01

**Authors:** S. Hejr, M. H. Karimi, S. Sabet, B. Mohammadi, S. Nikeghbalian, B. Geramizadeh, R. Yaghobi

**Affiliations:** ***Transplant Research Center, Shiraz University of Medical Science, Shiraz, Iran***

**Keywords:** Interleukin-18, Polymorphism, Liver transplantation, Graft rejection

## Abstract

Background: Cytokines and co-stimulatory molecules are important factors determining the outcome of transplantation.

Objective: To investigate the effect of IL-18 and CD40 gene polymorphisms on the outcome of liver transplantation.

Methods: 150 liver transplant recipients were included in this study. Alleles and genotypes frequencies for IL-18 (rs1946519) and CD40 (rs1883832) were determined in 28 acutely rejected (AR group) and 122 non-acutely rejected (non-AR group) liver transplant recipients. IL-18 and CD40 gene polymorphisms were evaluated by PCR-RFLP methods.

Results: There were no significant associations between IL-18 and CD40 polymorphism with acute rejection in liver transplant patients. IL-18TT and TG genotypes had a significant association with rejection in women compared to men. After grouping the liver recipients according to living *vs* cadaver donors, a significant association was found between CC genotype of CD40 and rejection in male living donor recipients. IL-18 TG genotype had a significant association with rejection in female cadaver donor recipients.

Conclusion: There is no correlation between all genotype and alleles of IL-80 and CD40 polymorphism and the outcome of liver transplantation. However, gender and type of donor affect the correlation between all genotype and alleles of IL-18 and CD40, and the outcome of liver transplantation.

## INTRODUCTION

Liver transplantation is the standard treatment for end-stage liver failure. Despite improvements in immunosuppressive therapy, acute rejection of the transplant remains an important cause of morbidity and late graft loss in patients undergoing liver transplantation. T-cell mediated immune response plays a critical role in the transplantation outcome [1]. For T-cell full activation, three separate signals are needed. The first signal comes through T-cell receptor and HLA. The second signal is delivered by T-cell co-stimulatory molecule, CD28, and the third signal is cytokines. 

Cytokines are a broad and loose category of small proteins that are important in cell signaling. They are released by cells and affect the behavior of other cells and sometimes the releasing cell, itself [2]. Naive T-cells under different cytokine milieu differentiate to various subtypes. In transplantation models, the Th1 cytokine profile often associates with allograft rejection, while the Th2 profile favors the acquisition of tolerance and stable graft survival [3]. Interleukin-18 (IL-18) is an important proinflammatory cytokine, member of the IL-1 cytokine family, which has been shown to exert innate and acquired immune responses [4, 5]. IL-18 is expressed by a wide range of immune cells and has been found to have multiple biological functions. The IL-18 has recently been shown to be a pleiotropic cytokine that can mediate both Th1- and Th2-driven immune responses [6]. IL-18 is associated with several human diseases including rheumatoid arthritis [7], and inflammatory bowel disease [8, 9]. In addition, several polymorphisms within the IL-18 promoter gene have been associated with different inflammatory and autoimmune diseases such as rheumatoid arthritis [10], systemic lupus erythematosus [11] and sub-acute sclerosing panencephalitis [12]. Also, in one study, it was shown that the -137GG genotype of the IL-18 gene, encoding higher IL-18 production, seems to be associated with allograft rejection and may be a useful marker of its risk in renal transplant recipients [13]. There are several studies on relation between IL-18 SNPs and outcome of transplantation. In one study, no significant differences in genotype and allele frequencies of (G137C) SNP were observed between the renal transplant recipients and the controls. However, the frequency of GG genotype at this position was significantly increased in patients with acute rejection compared to those without. Furthermore, they found that patients with GG genotype had significantly higher IL-18 serum levels compared to other genotypes [14]. Kolesar and colleagues evaluated the clinical significance of IL-18 gene SNPs at positions -607 A/C and -137 C/G in patients after kidney transplantation. They showed that the C allele at positions -607, which contributes to higher IL-18 expression, is more frequent in patients with delayed onset of kidney allograft function [15]. The co-stimulatory molecules deliver the second signal for T-cell full activation [16]. The CD40 is a co-stimulatory molecule that is a member of the tumor necrosis factor receptor (TNF-R) family; it is a surface receptor best known for its capacity to initiate multifaceted activation signals in normal B cells and dendritic cells (DCs) [17]. CD40 has been implicated in participating in many human diseases, particularly autoimmune diseases as well as the initiation and maintenance of inflammation triggered by infections through interaction with its ligand CD154 [17, 18]. Recently, genome-wide association studies revealed an association at the CD40 locus with rheumatoid arthritis [19] and multiple sclerosis [20]. Multiple candidate gene studies have also identified and validated the association of SNPs in CD40 with several autoimmune diseases including Graves’ disease [21], rheumatoid arthritis [22], and multiple sclerosis [23] but there were no study on CD40 gene polymorphisms and outcome of transplantation. Due to the role of functional CD40 and IL-18 polymorphisms in acute rejection and taking into account the importance of IL-18 and CD40 in T cell activation and important role of T cell activation in allograft rejection, we conducted this study to investigate the association of SNPs in the genes of IL-18 and CD40 with allograft function in liver transplant recipients.

## MATERIALS AND METHODS:

Patients

A total of 150 liver transplant recipients who had undergone surgery at Namazi hospital, Shiraz, Iran, was consecutively recruited from 2005 to 2009. Their age ranged from 1 to 66 years. Non-rejected transplant patients were considered as the control group. All of the patients were Iranian and had transplant operations at the Transplant Center of Namazi Hospital affiliated to Shiraz University of Medical Sciences. The study was approved by the Ethics Committee of Shiraz University of Medical Sciences. In this study, the patients were divided into two groups according to the presence or absence of acute rejection episodes. The demographic data was shown in Table 1. Acute rejection episodes were identified by an expert gastroenterologist team based on the accepted criteria such as increased serum levels of liver enzymes and total serum bilirubin level in the absence of biliary problems, histological findings after biopsy of the liver, and clinical and biochemical response to high-dose steroids [24]. The routine immunosuppression regimen consisted of tacrolimus or cyclosporine with mycophenolate mofetil and steroids. Drug dosage was adjusted to maintain target therapeutic blood levels of 200 ng/mL for CsA (5 mg/kg/day) or 10 ng/mL for tacrolimus. Donors were selected based on their ABO blood group compatibility. HLA matching was routinely not done for liver transplant patients.

**Table 1:** Demographic characteristics of liver transplant patient

Characteristic	Rejected patients group, n (%)	Non rejected group group, n (%)
Gender		
Male	16 (54)	54 (44)
Female	12 (43)	68 (56)
Type of transplant		
Cadaver	24 (86)	109 (89)
Living	4 (14)	13 (11)
Age	36.0±15.0	32.4±13.1

DNA extraction

The buffy coat of the whole blood from patients and control groups was made available from the sample bank of Shiraz Transplant Research Center. Genomic DNA was extracted from the buffy coat, using a QIAamp DNA Mini kit (Qiagen, Germany) according to the manufacturer’s instructions.

Genotyping analysis 

The SNPs of the study genes, IL-18 (-656G/T, rs1946519) and CD40 (C/T, rs1883832), were evaluated by PCR-RFLP methods using a thermal cycler (Techne, Genius, UK) as previously described [25, 26]. The PCR products were digested by restriction enzyme and the amplified products were monitored by agarose gel electrophoresis and ethidium bromide staining. Primers, fragment sizes and restriction enzymes are summarized in Table 2.

**Table 2:** The primers, fragment size and restriction enzymes for the IL-18 and CD40

Locus	Primers	Fragment sizes (bp)	Restriction enzymes
CD40-1C/T (rs1883832)	Forward primer: GAAACTCCTGCGCGGTGAAT Reverse primer: CCTCTT CCCCGAAGTCTTCC	CC133+74+85 CT 133+74+85+207 TT207+85	Sty1
IL-18(G-656T) (rs1946519)	Forward primer: AGGTCAGTCTTTGCTATCATTCCAGG Reverse primer: CTGCAACAGAAAGTAAGCTTGCGGAGAGG	TT 120 GT 120+96+24 GG 96+24	Mwo I

Statistical analysis

Allele and genotype frequencies were calculated in patient and control subjects by direct gene counting. Statistical evaluation was carried out using SPSS^®^ for Windows^®^ ver 16 (SPSS Inc, Chicago, IL, USA), and Epi Info (CDC, Atlanta, USA) software. The frequencies of the alleles/genotypes were compared in cases and controls by χ^2^ test or Fisher’s exact test when appropriate. Odds ratios and 95% confidence intervals (CIs) for relative risks were calculated. A p value <0.05 was considered statistically significant. All reported p values were two-tailed. Hardy-Weinberg equilibrium of the studied alleles was evaluated by Arlequin ver 3.1.1.

## RESULTS

Of 150 studied recipients, 46.7% were male and 53.3% were female. Male to female ratio was 16/12 (1.33) in those with rejection and 54/68 (0.79) in those without. Alleles and genotypes frequencies for IL-18 (-656G/T, rs1946519) and CD40 (C/T, rs1883832) were determined in 28 acutely rejected and 122 non-acutely rejected liver transplant recipients. None of the genotypes was in Hardy-Weinberg equilibrium in the studied groups of patients. Armitage’s trend test was used to check the association of genotypes with acute rejection whenever the Hardy-Weinberg equilibrium did not meet. However, after Bonferroni correction, no significant association was found between the studied alleles and genotypes with the transplant outcome. No significant associations was found between IL-18 and CD40 polymorphisms with acute liver graft in those with acute rejection and those without (Table 3). IL-18 TG genotype showed a significant association (p=0.02) with rejection in women compared to men. 

**Table 3:** The frequencies of CD40 and IL-18 genotypes and alleles in liver transplant patients with and without acute rejection

SNP (rs)	Genotype	With acute rejection			Without acute rejection			p_1 _value (OR)	p_2 _value (OR)	p_3 _value (OR)
		Male n (%)	Female n (%)	Total n (%)	Male n (%)	Female n (%)	Total n (%)			
IL-18 (G-656T) (rs1946519)	TT	4 (25)	0 (0)	4 (14)	9 (16)	14 (21)	23 (18.9)	0.45 (1.67)	0.08 (0.00)	0.57 (0.72)
	TG	10 (63)	12 (100)	22 (79)	44 (39)	47 (69)	91 (74.6)	0.38 (0.11)	0.02* (ND)	0.65 (1.25)
	GG	2 (13)	0 (0)	2 (7)	1 (44)	7 (10)	8 (6.6)	0.64 (7.57)	0.24 (0.00)	0.91 (1.10)
	T allele	18 (56)	12 (50)	30 (54)	62 (36)	75 (55)	137 (56.1)	0.90 (0.95)	0.64 (0.81)	0.72 (0.90)
	G allele	14 (44)	12 (50)	26 (46)	46 (64)	61 (45)	107 (43.9)			
CD40 (1C/T) (rs1883832)	TT	2 (13)	1 (8)	3 (11)	5 (9)	8 (12)	13 (10.7)	0.70 (1.40)	0.68 (0.72)	0.99 (1.01)
	TC	10 (63)	6 (50)	16 (57)	38 (70)	43 (63)	81 (66.4)	0.55 (0.70)	0.38 (0.58)	0.35 (0.67)
	CC	4 (25)	5 (45)	9 (32)	11 (20)	17 (25)	28 (23.0)	0.0.69 (1.30)	0.23 (2.14)	0.30 (1.59)
	T allele	14 (44)	8 (33)	22 (39)	48 (44)	59 (43.4)	107 (43.9)	0.94 (0.97)	0.35 (0.65)	0.53 (0.83)
	C allele	18 (56)	16 (67)	34 (61)	60 (55.6)	77 (56.6)	137 (56.1)			

p_1_ value indicates the difference between male patients with and without rejection.

p_2_ value indicates the difference between female patients with and without rejection.

p_3_ value indicates the difference between patients with and without rejection.

* Considered significant with P-value threshold of 0.05. In genotypes, each P-value is the result of comparing corresponding row with the sum of other rows.ND:not defined" OR :Odd ratio

In the present study, 11.3% of the recipients received the graft from living donors while 88.7% took their grafts from cadaver donors. A significant association (p=0.008) was found between CC genotype of CD40 and rejection in male living donor recipients (Table 4). IL-18 TG genotypes also had a significant association (p=0.04) with rejection in female cadaver donor recipients (Table 5).

**Table 4:** The frequencies of IL-18 and CD40 genotypes and alleles in liver transplant patients received allograft from living patients.

SNP(rs)	Genotype	With Acute Rejection			Without Acute Rejection			p_1_ value (OR)	p_2_ value (OR)	p_3_ value (OR)
		Male n (%)	Female n (%)	Total n (%)	Male n (%)	Female n (%)	Total n (%)			
IL-18 (G-656T) (rs1946519)	TT	0 (0)	0 (0)	0 (0)	2 (33)	2 (29)	4 (31)	0.49 (0.00)	0.30 (0.00)	0.20 (0.00)
	TG	1 (100)	3 (100)	4 (100)	4 (67)	5 (71)	9 (69)	0.49 (0.00)	0.30 (ND)	0.20 (ND)
	GG	0 (0)	0 (0)	0 (0)	0 (0)	0 (0)	0 (0)			
	T allele	1 (50)	3 (50)	4 (50)	8 (67)	9 (64)	17 (65)	0.64 (0.50)	0.55 (0.56)	0.43 (0.53)
	G allele	1 (50)	3 (50)	4 (50)	4 (33)	5 (36)	9 (64)			
CD40 (1C/T) (rs1883832)	TT	0 (0)	0 (0)	0 (0)	1 (17)	1 (14)	2 (16)	0.65 (0.00)	0.49 (0.00)	0.40 (0.00)
	TC	0 (0)	2 (67)	2 (50)	5 (83)	4 (57)	9 (69)	0.08 (0.00)	0.77 (1.50)	0.48 (0.44)
	CC	1 (100)	1 (33)	2 (50)	0 (0)	2 (29)	2 (15)	0.008* (ND)	0.88 (1.25)	0.15 (5.50)
	T allele	0	2 (33)	2 (25)	7 (58)	6 (46)	13 (50)	0.12 (0.00)	0.69 (0.67)	0.21 (0.33)
	C allele	2 (100)	4 (67)	6 (75)	5 (42)	8 (57)	13 (50)			

**Table 5:** The frequencies of IL-18 and CD40 genotypes and alleles in liver transplant patients received allograft from cadaver patients.

SNP(rs)	Genotype	With Acute Rejection			Without Acute Rejection			p_1_ value	p_2_ value	p_3_ value
		Male n (%)	Female n (%)	Total n (%)	Male n (%)	Female n (%)	Total n (%)			
IL-18 (G-656T) (rs1946519)	TT	4 (27)	0 (0)	4 (17)	7 (15)	12 (20)	19 (17.4)	0.28 (2.13)	0.14 (0.00)	0.92 (0.95)
	TG	9 (60)	9 (100)	18 (75)	40 (83)	42 (69)	82 (75.2)	0.05* (0.30)	0.04* (ND)	0.98 (0.99)
	GG	2 (13)	0 (0)	2 (8)	1 (2)	7 (11)	8 (7.3)	0.07 (7.23)	0.28 (0.00)	0.86 (1.15)
	T allele	17 (57)	9 (50)	26 (54)	54 (56)	66 (54)	120 (5.5)	0.96 (1.02)	0.74 (0.85)	0.91 (0.97)
	G allele	13 (43)	9 (50)	22 (46)	42 (44)	56 (46)	98 (45.0)			
CD40 (1C/T) (rs1883832)	TT	2 (13)	1 (11)	3 (13)	4 (8)	7 (11)	11 (10.1)	0.56 (1.69)	0.97 (0.96)	0.72 (1.27)
	TC	10 (67)	4 (44)	14 (58)	33 (69)	39 (64)	72 (66.1)	0.87 (0.91)	0.26 (0.45)	0.47 (0.72)
	CC	3 (20)	4 (44)	7 (29)	11 (23)	15 (25)	26 (23.9)	0.81 (0.84)	0.21 (2.45)	0.58 (1.31)
	T allele	14 (47)	6 (33)	20 (42)	41 (43)	53 (43.4)	94 (43.1)	0.70 (1.17)	0.41 (0.65)	0.85 (0.94)
	C allele	16 (53)	12 (67)	28 (58)	55 (57)	69 (56.6)	124 (56.9)			

P_1_ value=indicate the difference between reject and non-reject group in male Cadaver patients.

P_2_value= indicate the difference between reject and non-reject group in Female Cadaver patients.

P_3_ value= indicate the difference between reject and non-reject group in Cadaver patients.

* Considered significant with P-value threshold of 0.05. In genotypes, each P-value is the result of comparing corresponding row with the sum of other rows.ND:not defined" OR :Odd ratio

## DISCUSSION

Cytokines and co-stimulatory molecules are important factors determining the outcome of transplantation. Since the host ability in cytokine production and expression of co-stimulatory molecules may be affected by gene polymorphisms, the objective of the present study was to investigate the effect of IL-18 (-656G/T, rs1946519) and CD40 (C/T, rs1883832) gene polymorphisms on the outcome of liver transplantation. We found no significant associations between IL-18 and CD40 polymorphisms with acute liver graft rejection. IL-18TG genotype showed a significant association with rejection in women compared to men. Moreover, a significant association was observed between CC genotype of CD40 and rejection in male living donor recipients, and between IL-18 TG genotype and rejection in female cadaver donor recipients. To the best of our knowledge, there is no published article on association between IL-18 (-656 G/T) SNP and outcome of liver transplantation. Therefore, it was impossible to compare our results with others’. Nonetheless, there are several studies on the relationship between other IL-18 SNPs and the outcome of kidney transplantation. For example, Mittal, *et al*, investigated the association between interleukin-18 SNPs at positions 607A/C and 137C/G and kidney allograft survival in India. They showed protective association between the inheritance of CC genotype and C allele at position 137 of IL-18 and allograft rejection [27]. Kolesar and colleagues evaluated the clinical significance of IL-18 gene SNPs at positions -137 C/G and -607 A/C in patients after kidney transplantation. They showed that the C allele at positions -607, which contributes to higher IL-18 expression, is more frequent in patients with delayed-onset kidney allograft function [28]. In 2014, Bagheri, *et al*, showed that there were no significant associations between IL-18 polymorphisms and acute kidney allograft rejection. Also, they showed that after stratification of the recipients according to living *vs* cadaver donors, or according to their gender, there were no significant associations between the gene polymorphisms and acute rejection [29]. In addition, after categorization of liver recipients according to their gender, IL-18 TG genotypes showed a significant association with rejection in female patients compared to males. Moreover, TG genotype of IL-18 is a sex-dependent genetic risk factor for the development of acute rejection. This subject, however, needs to be studied in a different population. We could not find any study on IL-18 gene polymorphisms and gender in these patients. So, it was impossible to compare our results with others’. 

We could not find any data on the effect of sex hormone on CD40 and IL-18. Recently, genome-wide association surveys have identified the association of the SNPs in CD40 locus with several autoimmune diseases including multiple sclerosis, Graves’ disease and rheumatoid arthritis [30-32]. Chen, *et al*, showed no association between the rs4810485 and rs1883832 polymorphisms of CD40 with Fuchs’ uveitis syndrome in a Han Chinese population [33]. In another study, it was shown that there were no significant associations between CD40 polymorphisms and acute kidney allograft rejection. Also, after grouping the kidney recipients according to living *vs* cadaver donors or according to their gender, there were no significant associations between the above-mentioned gene polymorphisms and acute rejection [29]. We could not find any study on CD40 gene polymorphisms and outcome of liver transplantation. So, it was impossible to compare our results with those of others.

The significant associations we observed between IL-18 and CD40 allelic polymorphisms and acute liver rejection after gender classification showed a sex-dependent genetic risk factor for development of acute rejection. This subject, however, needs to be studied in other populations too.
